# Ethyl Chloride as a General Anæsthetic

**Published:** 1903-11-28

**Authors:** 


### ETHYL CHLORIDE AS A GENERAL ANÆSTHETIC.

Ethyl Chloride has now had a sufficiently extended trial as a general anæsthetic to allow of fairly satisfactory deductions being made with regard to the advantages and disadvantages which attend its use. T. D. Luke,[1] in a very instructive paper, summarises our present knowledge of its properties and uses. He points out that ethyl chloride was used to produce general anæsthesia so far back as 1851 by Snow, and later from time to time it received some attention from various anæsthetists, but it fell into disfavour as a general anæsthetic for some reason or other until in 1895 Carlson again directed attention to its properties. Since that time the drug has been very widely used in place of nitrous oxide, ether and chloroform.

Mr. Luke states that when inhaled ethyl chloride causes quickening and deepening of the respirations, with at first a slight decrease and then an increase of the cardiac pulsations; arterial pressure is raised, and the normal vascular tone stimulated during narcosis. If an adequate amount of air be not admitted during the course of the narcosis, the arterial pressure will soon fall, and respiration and then circulation become arrested. The anæsthetic is absorbed and eliminated with extraordinary rapidity, and this is a great factor for safety. There is practically no possibility of a cumulative effect. The first symptom of overdose is respiratory spasm, followed by arrest. The slowing or cessation of breathing results in decrease or cessation of absorption, while the commencement of artificial respiration brings back the patient to the normal. He also mentions the important fact that ethyl chloride keeps well, and has no tendency to underge decomposition or chemical change.

The production of anæsthesia is very rapid, and, if the patient takes deep breaths from the start, may be complete in 25 seconds. The average time is a little less than a minute, and after the inhaler has been removed the anæsthesia lasts on the average about 70 seconds. There are few unpleasant after-effects, the most common being headache and nausea, or vomiting. The tendency towards vomiting is dependent mainly upon the condition of the stomach, and an interval, if possible, of three or four hours is desirable between the last meal and the taking of the anæsthetic.

With regard to the death-rate of ethyl chloride anæsthesia Geitz's statistics are quoted, in which only one death was found in over 16,000 cases.

Mr. Luke thinks there is a wide field open for chloride of ethyl anæsthesia. Thus it is admirably adapted for dental work and for such short operations on the mouth and nose as the removal of tonsils and adenoids, polypi and spurs on the septum. The patient may be safely placed in the sitting posture, if the surgeon wishes, and the duration of anæsthesia and the recovery to consciousness may be regulated to within a few seconds. Again, young children and old people, in whom the use of nitrous oxide is frequently impossible or undesirable, take ethyl chloride remarkably well. As a preliminary to the use of chloroform it is also valuable owing to the rapidity with which consciousness is lost, and the consequent elimination of the dangerous struggling stage met with when chloroform is used by itself. Chloride of ethyl is, moreover, free from the pungency and unpleasantness of ether.

There are various forms of proprietary preparations on the market whose efficacy depends in the main upon ethyl chloride, but there are disadvantages in such mixtures, and it is better to rely on the pure compound manufactured by a reliable firm. Summarising the advantages of chloride of ethyl as an anæsthetic, Mr. Luke states that (1) that it is very rapid in action and pleasant to inhale; (2) the duration of anæsthesia compares very favourably with that afforded by nitrous oxide; (3) it causes no change of colour or cyanosis under normal conditions; (4) the administration is very simple in technique; (5) the after effects are slight, if there are any; (6) it is by no means expensive, but is cheaper than nitrous oxide; (7) it is especially useful when the patient is very young, very old, or anæmic; (8) it is extremely portable—a flask with sufficient for ten to twelve cases being readily carried in the breast pocket; (9) it is much safer than chloroform, as safe as ether, and much better adapted for brief operations than either.

[1] Edinburgh Medical Journal, November, 1903.

